# Cryptic Diversity in *Paramecium multimicronucleatum* Revealed with a Polyphasic Approach

**DOI:** 10.3390/microorganisms10050974

**Published:** 2022-05-05

**Authors:** Maksim Melekhin, Yulia Yakovleva, Natalia Lebedeva, Irina Nekrasova, Liubov Nikitashina, Michele Castelli, Rosaura Mayén-Estrada, Anna E. Romanovich, Giulio Petroni, Alexey Potekhin

**Affiliations:** 1 Faculty of Biology, Saint Petersburg State University, 199034 Saint Petersburg, Russia; maksim.s.melekhin@gmail.com (M.M.); yakovleva.spbu@gmail.com (Y.Y.); ne-irina@yandex.ru (I.N.); liubanik11@gmail.com (L.N.); 2 Laboratory of Cellular and Molecular Protistology, Zoological Institute RAS, 199034 Saint Petersburg, Russia; 3 Centre for Culture Collection of Microorganisms, Saint Petersburg State University, 198504 Saint Petersburg, Russia; nalebedeva@yandex.ru; 4 Department of Biology and Biotechnology ‘Lazzaro Spallanzani’, University of Pavia, 27100 Pavia, Italy; michele.castelli@unipv.it; 5 Laboratorio de Protozoología, Facultad de Ciencias, Universidad Nacional Autónoma de México, Circuito Ext. s/núm. Ciudad Universitaria, Av. Universidad 3000, Coyoacán, Ciudad de Mexico 04510, Mexico; rme2@ciencias.unam.mx; 6 Center for Molecular and Cell Technologies, Saint Petersburg State University, 199034 Saint Petersburg, Russia; aromanovich@gmail.com; 7 Department of Biology, University of Pisa, 56126 Pisa, Italy; giulio.petroni@unipi.it

**Keywords:** ciliates, biogeography, multi-loci phylogenetic analysis, micronucleus, cryptic species, species concept in protists

## Abstract

*Paramecium* (Ciliophora) systematics is well studied, and about twenty morphological species have been described. The morphological species may include several genetic species. However, molecular phylogenetic analyses revealed that the species diversity within *Paramecium* could be even higher and has raised a problem of cryptic species whose statuses remain uncertain. In the present study, we provide the morphological and molecular characterization of two novel *Paramecium* species. While *Paramecium lynni* n. sp., although morphologically similar to *P. multimicronucleatum*, is phylogenetically well separated from all other *Paramecium* species, *Paramecium fokini* n. sp. appears to be a cryptic sister species to *P. multimicronucleatum*. The latter two species can be distinguished only by molecular methods. The number and structure of micronuclei, traditionally utilized to discriminate species in *Paramecium*, vary not only between but also within each of the three studied species and, thus, cannot be considered a reliable feature for species identification. The geographic distribution of the *P. multimicronucleatum* and *P. fokini* n. sp. strains do not show defined patterns, still leaving space for a role of the geographic factor in initial speciation in *Paramecium*. Future findings of new *Paramecium* species can be predicted from the molecular data, while morphological characteristics appear to be unstable and overlapping at least in some species.

## 1. Introduction

*Paramecium* O.F. Müller, 1773 is one of the most recognizable ciliates that has attracted attention already in the early studies of protozoology. Paramecia can be found all over the world, being important and, sometimes, an abundant component of microbial communities in freshwater ecosystems [1]. The easy identification of representatives of this genus, compared with many other ciliates, and its simple maintenance in laboratory conditions made *Paramecium* a unicellular model organism of choice in genetics, cellular, and molecular biology [2,3]. The systematics of *Paramecium* has always been of special interest. Many morphological species as well as reproductively isolated groups within them, *de facto* genetic species, such as the sibling species of the *P. aurelia* complex, have been described in the pre-molecular era [4,5]. Molecular studies led to a real breakthrough in the validation of some doubtful species [6,7,8], to the discovery of new species [9], and to the identification of several species with morphological peculiarities that were not so discernible [10,11]. The complex structure of many morphological species was also unraveled, confirming that almost each species includes several phylogenetic subgroups [12,13]. Such subgroups may correspond to reproductively isolated groups known as syngens, which, in fact, in most cases, are equivalent to young cryptic species [13,14].

The genus *Paramecium* is subdivided into six subgenera, which have no official taxonomic recognition: Chloroparamecium, Gigaparamecium, Viridoparamecium, Helianter, Cypriostomum, and Paramecium *sensu stricto* [8,9,10,15]. The former three are represented by one species each: *P. bursaria*, *P. gigas*, and *P. chlorelligerum*, respectively. Subgenera Helianter and, especially, Cypriostomum consist of a number of species that are difficult to diagnose [16,17]. The “classical” morphospecies of *Paramecium*, namely *P. caudatum*; *P. multimicronucleatum*; the *P. aurelia* complex, which includes 16 sibling species [2,18,19,20]; and the closely related *P. jenningsi* and *P. schewiakoffi* [20] belong to the subgenus Paramecium *s. str.* A cigar-shaped body, the relatively big size, a cytostome positioned at cell equator, and a cytoproct located at some distance from the posterior end of the cell are characteristic of the representatives of this subgenus [15]. Several other species, which should also be attributed to subgenus Paramecium s. str., were documented but cannot be considered valid due to incomplete characterization and only single findings. Most of them, such as *P. africanum*, *P. jankowskii*, *P. ugandae*, and *P. wichtermani*, were reported from less studied territories of Africa (see [16,21]). The special term “*Eucandidatus*” was coined to make a distinction between valid species and the provisional cryptic species status [11], and several cryptic species assigned to this subgenus were recently described from Europe (“*Eucandidatus* P. germanicum” [11]) and South America (“*Eucandidatus* P. brazilianum” [11] and *P. grohmannae* [22]). Molecular phylogenetic studies revealed that the species diversity within *Paramecium* could still be higher than previously known, as the representatives of some branches of the trees inferred from the 18S rRNA gene [11,23,24] have never been studied morphologically. For example, molecular phylogenetic studies always revealed two separate branches within well-known species *P. multimicronucleatum* [11,12,22], but a comparative morphological analysis has never been accomplished for the strains representing both of these clades.

In the present study, we provide the morphological and molecular characterization of two novel species belonging to the subgenus Paramecium *s. str.* Both species may be morphologically disguised as *P. multimicronucleatum* but could be predicted from molecular phylogenetic data.

## 2. Materials and Methods

### 2.1. Sampling, Strain Choice, and Culture Maintenance

In total, 35 *Paramecium* strains originating from Europe, Asia, and North America were used in this study (see Table 1). Paramecia were initially detected under stereomicroscope Nikon SMZ 800 (Nikon, Tokyo, Japan) in water samples taken from natural waterbodies, and several cells from each population were isolated separately into microaquaria. The established clonal cultures were maintained at 18–21 °C on lettuce medium bacterized the day before use with *Enterobacter cloacae* and supplemented with 0.8 mg/L of β-sitosterol (Merck, Darmstadt, Germany), as described earlier [2]. The synchronization of cultures aiming to obtain sexual reactivity and observation of sexual processes were achieved by daily re-isolations [3]. All currently extant strains are available upon request from the RC CCM collection (World Data Centre for Microorganisms, RN 1171), Saint Petersburg State University, Saint Petersburg, Russia.

Using DIC microscopy, we selected a set of *Paramecium* sp. strains, with cells corresponding to the size, shape, and general appearance of MICs of *P. multimicronucleatum*. This morphological species is characterized by big cigar-shaped cells (live specimens are more than 220 µm in length and more than 60 µm in width), with roundish anterior ends and pointed posterior ends. The cells normally possess two CVs with 6–9 canals and 1 pore each. The macronucleus (MAC) is single; oval-shaped; and medium sized, 55 × 20 µm. Autogamy, which is a regular self-fertilization process followed by fragmentation of the old MAC typical for the species of the *P. aurelia* complex, was never reported for *P. multimicronucleatum*. The tiny vesicular MICs are numerous compared with most other *Paramecium* species (in the original species description, from 2 to 6 [25]; in [26,27], from 2 to 5; and in [4], typically 3) and usually are located in proximity to the MAC. Other *Paramecium* species, with comparable cell dimensions, such as *P. caudatum*, *P. schewiakoffi*, *P. jenningsi*, and some representatives of the *P. aurelia* complex, have distinct types of MIC and may easily be discriminated from *P. multimicronucleatum* by its morphological characteristics.

### 2.2. DIC Microscopy and Stainings

Initial live cell observations were made with differential interference contrast (DIC) microscopy using a Nikon Eclipse Ni microscope equipped with a DS-Fi3 camera (Nikon, Tokyo, Japan). The mechanical microcompressor Commodore [28] was used for immobilization, observation, and imaging of live specimens. We observed the cytological features important for quick species identification in *Paramecium*, namely cell size and shape; size, number, and structure of micronuclei; structure of contractile vacuoles; and signs of nuclear rearrangements [16]. The Feulgen staining procedure and silver nitrate impregnation after Champy’s fixation following Chatton and Lwoff modified protocol [29,30] were employed for detailed morphometric analysis, visualization of the cortex, and nuclear apparatus peculiarities. Morphometric measurements were taken from at least 30 stained cells of each strain studied. All measurements were made using either NIS-Elements software (Nikon, Tokyo, Japan) or the FiJi ImageJ program (Babraham institute, UK).

### 2.3. Molecular Identification of Paramecium Strains

All studied Paramecium strains were subjected to sequencing of at least one of three genetic markers, namely the 18S rRNA gene, the ITS1-5.8S-ITS2 region (further on referred to as ITS region), and the mitochondrial cytochrome C oxidase subunit I (COI) gene. The total cell DNA was extracted from 50–100 cells of each strain using the GenElute Mammalian Genomic DNA Purification Kit (Sigma-Aldrich, Darmstadt, Germany) according to the protocol «Genomic DNA from tissue» or NucleoSpin Tissue kit (Macherey-Nagel, Duren, Germany). The PCRs were performed in Mastercycler nexus (Eppendorf, Hamburg, Germany) using Encyclo polymerase (Evrogen, Moscow, Russia). The primers used for PCRs and sequencing are listed in Table S1. Oligonucleotides were synthesized by Evrogen (Moscow, Russia). The amplification of the 18S rRNA gene and of the ITS region was generally performed as described earlier [31]. The 761 bp long COI gene sequences were amplified as described in [32]. The annealing temperature and the number of PCR cycles were different depending on the marker (COI gene—56 °C, 35 cycles; 18S rRNA gene—65 °C, 39 cycles; and ITS region—65 °C, 35 cycles). The cloning of the PCR products was performed to obtain pure ITS region sequences of some strains. The CloneJET Kit (Thermo Scientific, Waltham, MA, USA) was used for the insertion of PCR amplicons of the ITS region into plasmid pJET 1.2/blunt with prior blunting (following the standard sticky-end cloning protocol) and the transformation of XL10-Gold strain of *E. coli*-competent cells by temperature shock [33]. Transformed cells of *E. coli* were grown on the plates with LB medium supplemented with ampicillin (50 mg/mL) for positive selection. The check for recombinants was performed on 3–5 colonies from each plate via PCR screening following the standard protocol with pJET 1.2 Forward and pJET 1.2 Reverse Sequencing primers (Thermo Scientific, Waltham, MA, USA). The PCR products containing the insert were subjected to sequencing utilizing the primers used for the amplifying PCR. All PCR products were sequenced unpurified at the Center for Molecular and Cell Technologies (St Petersburg State University, Saint Petersburg, Russia).

### 2.4. Fluorescence In Situ Hybridization Assay

Two fluorescent oligonucleotides (Table S1) targeting highly conserved unique regions in the 18S rRNA sequence of strains representing two groups of *P. multimicronucleatum* were designed in silico and employed for fluorescence in situ hybridization (FISH) to discriminate between the strains of these groups. The probes were synthesized and labeled with Cy3 or FITC by Eurofins GMBH (Ebersberg, Germany). FISH experiments were performed at different formamide concentrations (0%, 15%, and 30%), and the signal was sharper at 15 % formamide. The cells were fixed on adhesion slides (Thermo Scientific Super Frost Plus, UK) by 4% paraformaldehyde for 5 min, then washed in distilled water for 10 min, dehydrated in ethanol, and hybridized with fluorescent probes as described before [34] at 46 °C. After hybridization, the cells were washed at 52 °C for 30 min twice and then covered with ProLong^®^ Gold antifade mountant with DAPI (Invitrogen, UK). All experiments included negative controls. No less than 20 cells were observed on each slide. All FISH observations were performed using a Nikon Eclipse Ni (Nikon, Tokyo, Japan) fluorescent microscope.

### 2.5. Molecular Phylogenetic Analysis

Nucleotide alignments were made in the MAFFT v.7 e-ins-i algorithm [35] and manually curated. We constructed conservative blocks with less stringent selection (smaller final blocks, gap positions within the final blocks, and less strict flanking positions allowed) for the 18S rRNA gene and ITS region alignments using Gblocks [36] implemented in SeaView v.5 [37]. The final alignment lengths were 1705 bp for the 18S rRNA gene, 1066 bp for the ITS region, and 761 bp for the COI gene. The alignments were analyzed in RAxML BlackBox v.8.2 [38]. We used a GTR model with a CAT approximation, and all parameters were estimated from data, with 500 bootstraps for the 18S rRNA gene, 600 for the ITS region, and 1000 for the COI gene. Bayesian consensus trees were constructed with MrBayes v.3.2.7a [39]. For each alignment, four separate runs with four chains for each with randomly generated starting trees were carried out for 10 M generations. The evolutionary model applied included a GTR substitution matrix with gamma-distributed rate variation across sites and a proportion of invariable sites. Trees were sampled every 1000 generations. The first 25% of samples from the cold chain were discarded as burn-in. All phylogenetic analyses were performed via the Cipres Science Gateway [40]. Visualization of phylogenetic trees was carried out with the ETE 3 Python package [41].

**Table 1:** Data on the strains used in the study.

Species	Strain Index	Geographic Origin and Year of Collection	Biotope	Morphological and Physiological Characteristics	NCBI Accession Numbers		
					COI	18S rDNA	ITS1-5.8S-ITS2
*Paramecium multimicronucleatum*	CyP5-3	Cyprus, Paralimni, 2016	ditch, 2‰	CL: 130.7 ± 10.9 µm; CW: 30.9 ± 4.1 µm; ML: 55.7 ± 13.8 µm; MW: 20.2 ± 3 µm; NM: 1 or 3; DMIC: 2.09 ± 0.34 µm; vesicular MIC; MIC location: freely in cytoplasm; NCVC: 6–7; NCR: 74 ± 9	OM401905	OM200731	
	ID1-13	India, Delhi, 2018	pond		OM401906	nd	nd
	MSA-5	Malta, San Anton Gardens, 2013	stone bowl		OM401907	OM200732	
	Ns2-16	Russia, Novosibirsk, 2002	creek		OM401908	OM200756	[12]
	Vv171-1	Russia, Vladivostok, 2007	pond		OM221497	OM200757	[12]
	Thk-16	Thailand, Phi Phi don, 2014	creek		OM401909	OM200733	
	R51-6	Mexico, Requena Lake, 2019	lake	CL: 149.3 ± 11 µm; CW: 27.8 ± 4.3 µm; ML: 51.1 ± 8.1 µm; MW: 16 ± 2.6 µm; NM: 1–3; DMIC: 4.31 ± 0.3 µm; vesicular MIC; MIC location: near MAC; NCVC: 5–8; NCR: 73 ± 8; Intrastrain conjugation (selfing) observed in some strains	OM401910	OM200734	
	SMM80-11	Mexico, San Miguel Almaya, 2019	lake		OM401911	nd	nd
	ChP10-2	Mexico City, Chapultepec, 2019	lake in the city park		OM401912	OM200735	
	K4-2	Mexico City, Cantera Oriente, 2019	pond		[19]	OM200736	
	L72-1	Mexico, Lerma, 2019	marsh		[19]	OM200737	
	MB2-5	Moldova, Bendery, 1996	river		[12]	OM200758	[12]
	Or4-3	Russia, Orenburg, 2015	wastewater stream		OM221498	OM200738	
	OmN-1	USA, Omaha, NE, 2018	Missouri river		OM401913	OM200739	
	SK6-3	Mexico, Sian Kaan, 2019	ditch		OM401914	OM200740	
	LB-2	Mexico, Bacalar, 2019	freshwater lagoon		OM401915	OM200741	
	ChP3-4	Mexico City, Chapultepec, 2019	lake in the city park	CL: 180.3 ± 13.9 µm; CW: 36.2 ± 3.9 µm; ML: 61 ± 7.5 µm; MW: 19 ± 2.7 µm; NM: 2; DMIC: 3.49 ± 0.46 µm; vesicular MIC; MIC location: freely in cytoplasm; NCVC: 6–8; NCR: 72 ± 7	OM401916	nd	nd
	E59-1B	Mexico, Endoh Lake, 2019	lake		OM401917	nd	nd
	IP2-1	Italy, Pisa, 2016	channel		OM401918	nd	nd
	ChP5-3	Mexico City, Chapultepec, 2019	lake in the city park		[19]	OM200742	
*Paramecium fokini* n. sp.	SMM81-1	Mexico, San Miguel Almaya, 2019	lake	CL: 155.6 ± 13.9 µm; CW: 27.6 ± 4,8 µm; ML: 54.6 ± 7.7 µm; MW: 16.4 ± 2.4 µm; NM: 1–3; DMIC: 4.1 ± 0.66 µm; vesicular MIC; MIC location: near MAC; NCVC: 6–8; NCR: 67 ± 8; Intrastrain conjugation (selfing) observed in some strains	[19]	OM200743	
	T42-1	Mexico City, Tlahuac, 2019	channel		[19]	OM200744	
	AB9-8	USA, Boston, 1994	pond		OM401919	OM200759	[12]
	CyL3-21	Cyprus, Larnaka, Aliki region, 2010	ditch, 1–2‰		OM401920	OM200745	
	PP-2	Russia, Pskov region, 2012	ditch		OM401921	OM200746	
	PL4-1	Portugal, Lisbon, 2019	concrete basin		OM401922	OM200747	
	OP13	Russia, Saint Petersburg, 1992	city pond		OM401923	OM200760	[12]
	FCB10-1	France, Corsica, Bastia, 2015	stream		OM221499	OM200748	
*Paramecium lynni* n. sp.	ShKm41	Russia, Kemerovo region, Shestakovo, 2008	river	CL: 133.7 ± 13.1 µm; CW: 31.9 ± 5.1 µm; ML: 38.6 ± 6.2 µm; MW: 15.8 ± 2.8 µm; NM: 1–3; DMIC: 3.55 ± 0.46 µm; ‘fried egg’ MIC; MIC location: freely in cytoplasm; NCVC: 6–8; NCR: 64 ± 8	OM401924	OM200749	
	HSG3-10	Russia, Saint Petersburg region, Peterhof, 2017	ditch		OM401925	OM200750	
	SD11-9	Russia, Saint Petersburg region, Sestroretsk, 2017	pond		OM401926	OM200751	
	PO16-1	Russia, Pskov region, Ostrov, 2019	pond		OM401927	OM200752	
	SP-1	Russia, Saint Petersburg region, Peterhof, 2019	pond		OM401928	OM200753	
*Paramecium caudatum*	Or4-4	Russia, Orenburg, 2015	wastewater stream	Cell size about 200 µm; single large compact MIC adjacent to MAC; 5–8 (more often 7) canals of CV; 1 pore per CV *	OM401929	OM200754	
	K5-2	Mexico City, Cantera Oriente, 2019	pond		[19]	OM200755	

CL—cell length; CW—cell width; ML—MAC length; MW—MAC width; NM—number of MICs; DMIC—diameter of MIC; NCVC—number of CV canals; NCR—number of ciliary rows. All morphometric measurements produced on Bouin-fixed cells. nd—not determined. * in agreement with the data from [16].

## 3. Results

### 3.1. Identification of the New Species Distant from Paramecium multimicronucleatum

**Figure 1:**
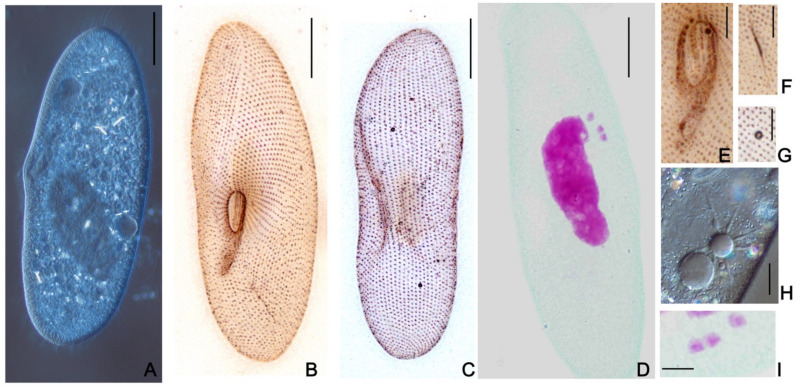
Morphological features of *Paramecium lynni* sp. n. (**A**) DIC live micrograph of a specimen. Silver nitrate impregnated cells: (**B**,**C**) ventro-lateral and dorso-lateral cell projections; (**D**) Feulgen stained specimen with three MICs; (**E**) buccal overture with buccal ciliature; (**F**) cytoproct region; (**G**) one pore characteristic per contractile vacuole. (**H**) the contractile vacuole DIC live micrograph. (**I**) micronuclei having specific “fried egg” appearance shown by the Feulgen stain. Scale bars: 20 µm (**A**–**D**) and 4 µm (**E**–**I**).

Traditionally, several morphological features are considered informative for defining the *Paramecium* species [16]: cell size and shape, type and number of micronuclei (MICs), the structure of contractile vacuole (CV), and the number of pores. Based on these characteristics, 33 strains were initially classified as plausible representatives of *P. multimicronucleatum*, though some of their features (Figure 1) might not fit the accepted species diagnosis [16]. The detailed morphological analysis was accomplished for all selected strains. In parallel, they were subjected to 18S rRNA gene sequencing, which is traditionally used to attribute *Paramecium* strains to certain morphological species, while the differences between sibling species or syngens were not resolved [32,42]. Two other loci, namely, the nuclear ITS region and the mitochondrial COI gene, were sequenced for most or all studied strains.

**Figure 2:**
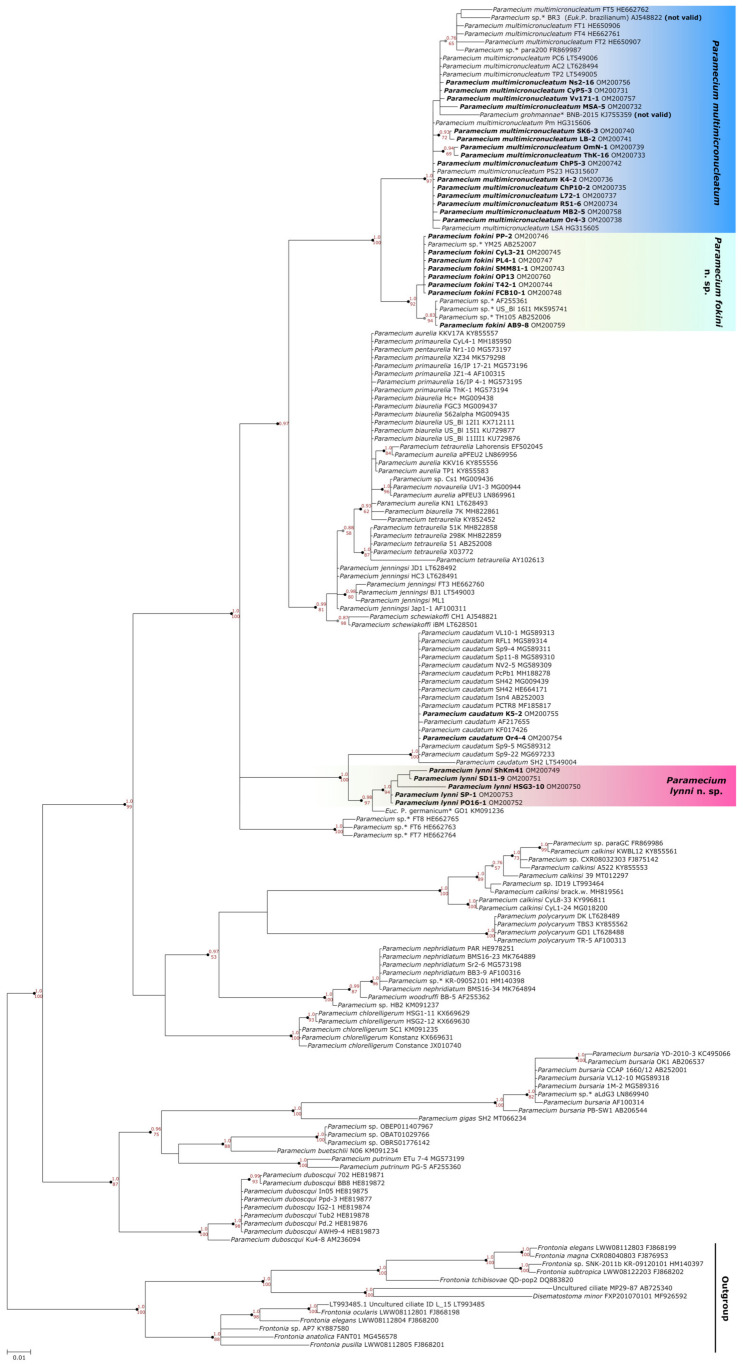
Phylogenetic position of three groups of *Paramecium multimicronucleatum*-like strains on the 18S rRNA gene tree. Numbers associated with the nodes represent a posterior probability from Bayesian inference (BI) and the bootstrap value from maximum likelihood (ML) analyses. Strains marked in bold were analyzed in this study. Asterisks (*) indicate the misidentified or non-identified at the species level strains in NCBI (see Table S3).

**Figure 3:**
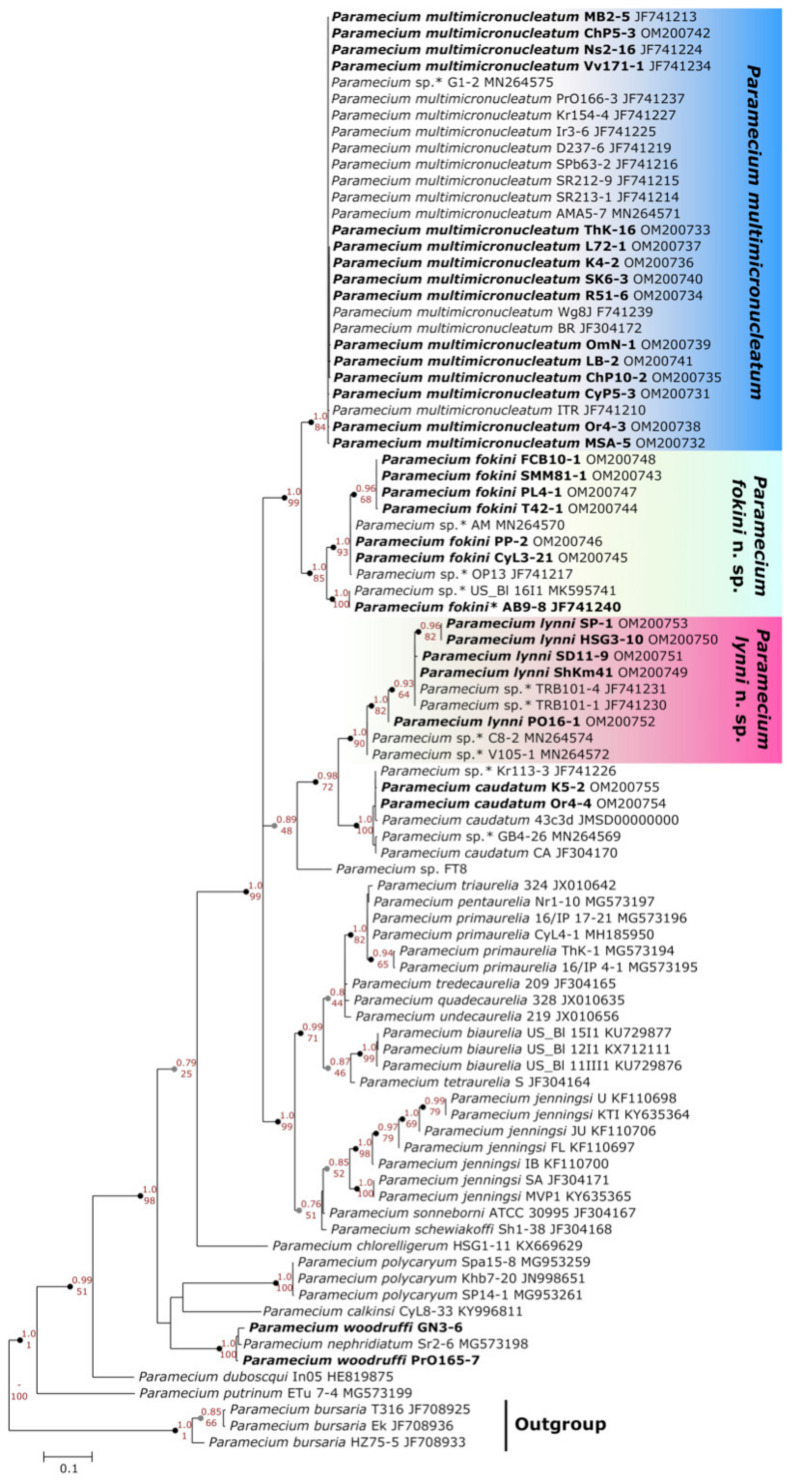
Phylogenetic position of three groups of *Paramecium multimicronucleatum*-like strains on the ITS region tree. Numbers associated with the nodes represent posterior probability from Bayesian inference (BI) and the bootstrap value from maximum likelihood (ML) analyses. Strains marked in bold were analyzed in this study. Asterisks (*) indicate the misidentified or non-identified at the species level strains in NCBI (see Table S3).

**Figure 4:**
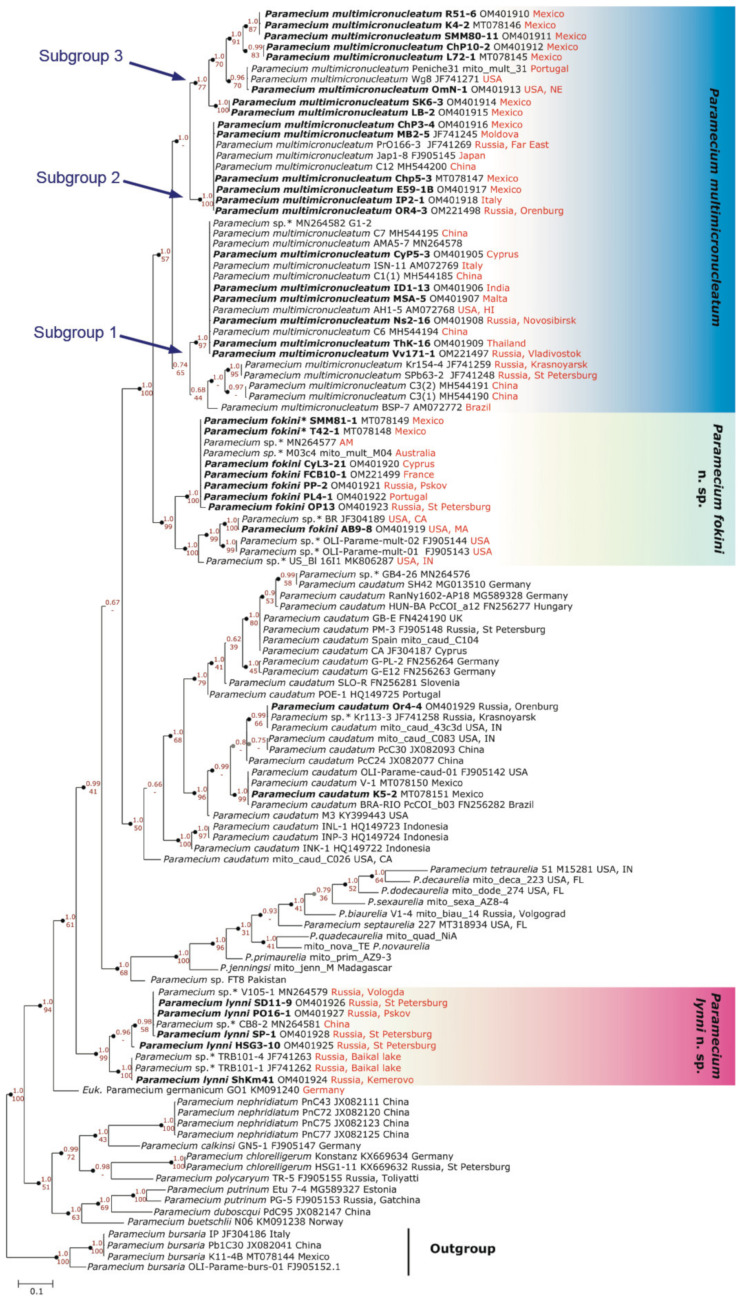
Phylogenetic position of three groups of *Paramecium multimicronucleatum*-like strains on the mitochondrial COI gene tree. Numbers associated with the nodes represent posterior probability from Bayesian inference (BI) and the bootstrap value from maximum likelihood (ML) analyses (only values of BI > 0.7 are shown). The symbol - indicates different architecture between ML and BI trees at the particular node. Strains marked in bold were analyzed in this study. Asterisks (*) indicate the misidentified or non-identified at the species level strains in NCBI (see Table S3). The arrows show three intraspecies subgroups within *Paramecium multimicronucleatum*.

All 33 strains we selected were split into three groups on the 18S rRNA gene phylogenetic tree of *Paramecium* (Figure 2). Two of them corresponded to two previously reported [11,12] subclades within *P. multimicronucleatum* (see below), while the third one formed by five strains together with “*Eucandidatus* P. germanicum” [11] unexpectedly formed a distant branch sister to *P. caudatum*. The identity of their sequences among each other and with the 18S rRNA gene of “*Eucandidatus* P. germanicum” reached 98.6–100%; with *P. caudatum*, it was 97.1–98.2%; and with *P. multimicronucleatum*, it was not higher than 95.2%. The phylogenetic analysis of the ITS region confirmed that these five strains clustered together with *P. caudatum* and “*Eucandidatus* P. germanicum” (Figure 3). In the COI gene topology, these strains, together with “*Eucandidatus* P. germanicum” branched basally in the Paramecium *s. str.* subgenus (Figure 4). Maximal identity with the *P. caudatum* strain COI gene sequences reached only 87%, while that with the sequences of the strains from the *P. multimicronucleatum* cluster was even less, maximum 85.5% (and just ~84% with “*Eucandidatus* P. germanicum”).

These five strains from distant parts of Russia, three from Saint Petersburg and its vicinity, one from the Pskov region in the northwestern part of Russia, and one from the Kemerovo region in Western Siberia, shared the same morphological peculiarities, which, though still similar to *P. multimicronucleatum*, all together merged into a different cell phenotype (Figure 1 and Table 1). First, all five strains had very peculiar MICs, which could not be classified as “vesicular” (i.e., small spherical nuclei where chromatin mass occupies the periphery, while in the center there is a Feulgen-negative “vesicle” [16]). These MICs, varying in number from strain to strain (one in PO16-1; two in ShKm41; and three in SD11-9, HSG3-10, and SP-1 strains), were bigger than typical vesicular nuclei and looked more similar to the “endosomal” type, where the chromatin body is separated from the nuclear envelope by a distinctive empty rim [16]. However, there was also still a non-pronounced “vesicle,” looking more like a dimple, in the middle of such MICs, and some chromatin was observed in Feulgen-stained cells in the space between the chromatin body and the nuclear envelope. In cells of these strains, there was no specific location of the MICs, but they could be found close to MAC or free in the cytoplasm. Their MACs were smaller (38 × 15 µm) and more roundish than in *P. multimicronucleatum*, which has an elongated MAC. The cells were at a size range of relatively small *P. multimicronucleatum* in length (133.7 ± 13.1 µm), but at the same time, they looked a bit wider (31.9 ± 5.1 µm). Both ends of the cells looked blunt. The number of cilia rows was 64 ± 8. The cytostome was located close to the cell equator, while the cytoproct could be found approximately midway between the buccal overture and the posterior end of the cell. Two contractile vacuoles always had one pore each, and the number of collecting canals was 6–8. We never achieved conjugation within the strain or between the cells of different strains. Autogamy was never observed.

Therefore, both morphological and molecular data confirmed that this group of strains represented a novel *Paramecium* morphological species. We named it *P. lynni* n. sp.

### 3.2. Two Groups Revealed within Paramecium multimicronucleatum Cluster

#### 3.2.1. Molecular Methods Suggest Two Cryptic Species within *P. multimicronucleatum*

Two groups of strains distinguished by comparison of the 18S rRNA gene sequence corresponded to two previously reported branches within *P. multimicronucleatum* morphological species. Twenty strains joined “group I”, and eight strains belonged to “group II”. The 18S rRNA gene sequence identity between the strains from these groups varied from 96.9% to 98.6%.

A comparison of the strains from both groups according to the other two marker sequences confirmed the consistency of the groups I and II, as the same strains dropped into the same clusters. The ITS region appeared to be a rather conservative marker, showing a certain not very pronounced divergence within both groups (Figure 3). The most discrete phylogeny was inferred from the COI gene, as at least three subgroups could be distinguished within group I and two defined subgroups were revealed within group II (Figure 4). The COI gene sequence difference between the strains from groups I and II varied from 6.9% to 12.2%.

**Figure 5:**
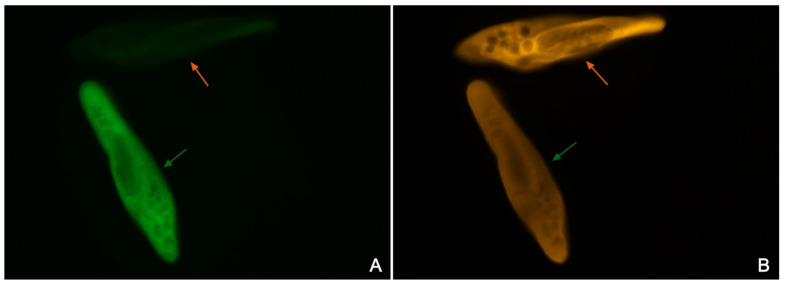
Discrimination of representatives of *P. multimicronucleatum* groups I and II by FISH: (**A**) mixed cells of both groups hybridized with Paramulti probe (green signal) specifically designed for group I; (**B**) mixed cells of both groups hybridized with Parafok probe (orange signal) specifically designed for group II. The cell of group I is marked with a green arrow, and that of group II is with an orange arrow. Paramulti probe appeared to be efficient at exclusively detecting representatives of strains belonging to group I (cell marked with an orange arrow on (**A**) remains almost invisible), while Parafok bound to cells of both groups.

The difference in the 18S rRNA gene sequence between the strains of groups I and II allowed us to design two probes for FISH, specifically matching unique sequences in the SSU rRNA of both groups (Table S2). The probe Paramulti specific to group I was labelled with FITC. The bright green hybridization signal was achieved after FISH with cells of the strains representing all three subgroups of group I, while it was almost invisible when the strains of the group II were used (Figure 5 and Figure S1) and never produced positive signals applied to cells of other *Paramecium* species. The Cy3-labeled probe Parafok designed specifically for group II strains, notwithstanding, appeared to also match rather efficiently cells of the strains belonging to group I (Figure 5 and Figure S1). Thus, only the probe Paramulti designed for group I allowed for faithful detection of strains belonging exclusively to this group.

The level of three marker sequences diverging, together with the possibility to distinguish strains belonging to two groups by FISH, allowed us to suggest the assignment of a cryptic species rank to groups I and II. As the group I strains satisfy the existing diagnosis of *P. multimicronucleatum* (see below), this name should be kept for it. We named the new species represented by the strains of group II as *Paramecium fokini* n. sp.

#### 3.2.2. Morphological Variability within *Paramecium multimicronucleatum*

A certain morphological variability among the strains belonging to two significantly divergent clades representing *P. multimicronucleatum* and *P. fokini* n. sp. might be expected. Thus, we performed a thorough comparison of the strains belonging to both species.

The morphological analysis of 12 strains belonging to *P. multimicronucleatum* and 7 strains belonging to *P. fokini* n. sp. brought a surprising result. As supposed by the species name, the *P. multimicronucleatum* characteristic feature is the presence of several MICs, at least two but more commonly three or four, and up to five [16]. We stained the cells of all examined strains by Feulgen no less than two times to be sure that the number of MICs does not vary from cell to cell of the same strain or change during the cell cycle. Normally, in the cells of the same strain, the number of MICs was constant and could serve as a strain characteristic. Curiously enough, we found that the number of MICs in the studied strains of both groups ranged from 1 to 3, and in particular, there were four *P. multimicronucleatum* strains and two *P. fokini* n. sp strains with a single MIC (Table 1).

**Figure 6:**
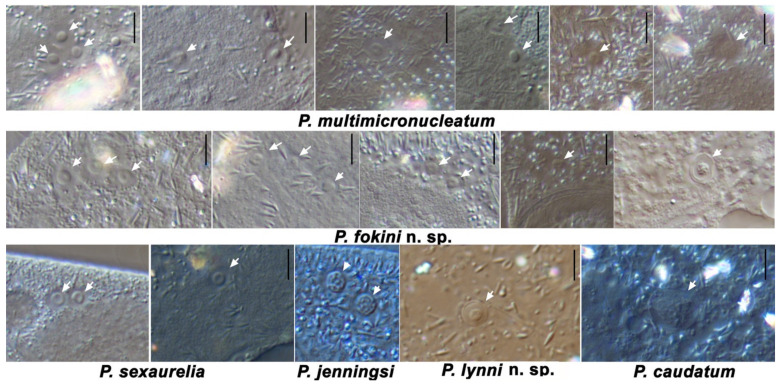
Variability in micronuclei shape and appearance of several Paramecium *s. str.* subgenus species. DIC microscopy. Micronuclei are marked with the arrows. Scale bar is 4 µm.

A vesicular MIC considered typical of *P. multimicronucleatum* looks different from classical vesicular MICs of the *P. aurelia* species (Figure 6). While the latter always have a pronounced vesicle in the middle of the chromatin body and MIC resembles a donut, the MICs of *P. multimicronucleatum* are in general smaller, and the vesicle may be invisible or well detected even in MICs of the same cell. The stage of cell cycle also probably influences the morphology of the MIC, as MICs may look different in the cells of the same strain (Figure S2). In the strains of *P. multimicronucleatum* and *P. fokini* n. sp., we registered many morphological variants of MICs (Figure 6). Some strains had regular vesicular MICs, and other strains had MICs where a vesicle was not visible at all, resembling MICs of *P. polycaryum* (Cypriostomum subgenus) or even small nuclei of compact type. Some strains had MICs with visible chromatin fibers in the middle, thus reminiscent of the chromosomal MICs of *P. jenningsi*.

**Figure 7:**
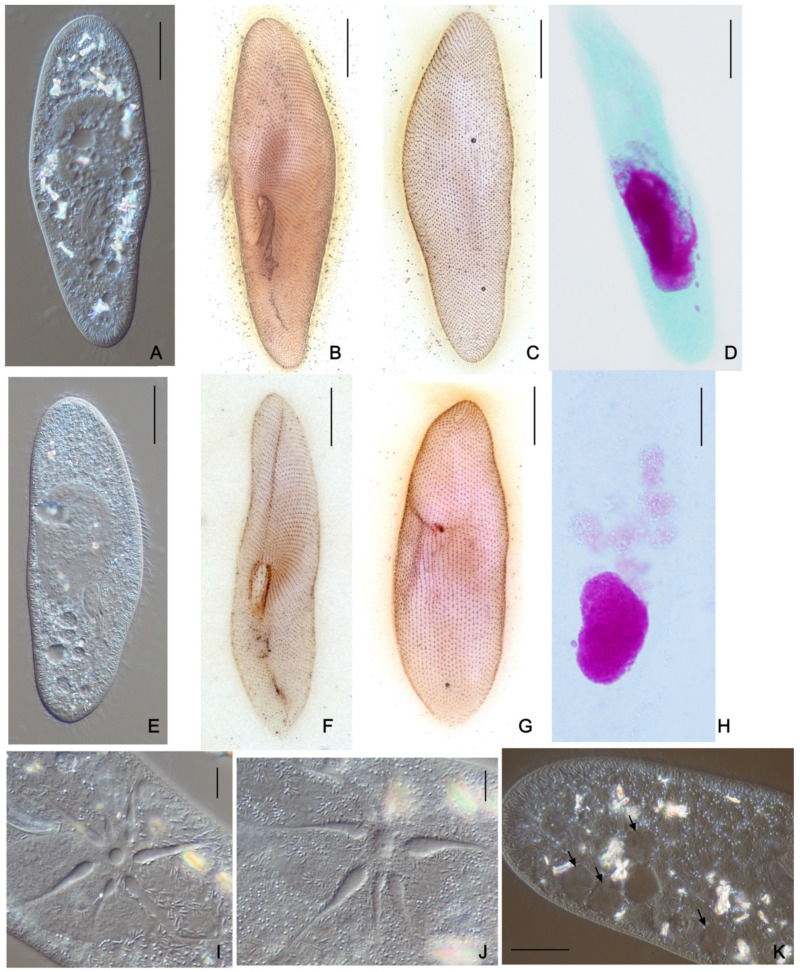
Morphological features of *Paramecium multimicronucleatum* and *P. fokini* n. sp. Strains of *P. multimicronucleatum*: (**A**) DIC live micrograph of a specimen. (**B**,**C**) Silver nitrate impregnated cells: ventro-lateral and dorso-lateral cell projections; (**D**) Feulgen stained specimen with three MICs. Strains of *P. fokini* n. sp.: (**E**) DIC live micrograph of a specimen. (**F**,**G**) Silver nitrate impregnated cells: ventro-lateral and dorso-lateral cell projections; (**H**) Feulgen stained specimen with two MICs. Contractile vacuoles of both species (**I**,**J**) are very similar. (**K**) DIC live micrograph showing the exconjugant cell of a strain of *P. fokini* n. sp.; four MAC anlagen are marked with the arrows. Scale bars: 20 µm (**A**–**D**) and 4 µm (**E**–**I**).

In general, there were no conspicuous morphological differences between the strains of *P. multimicronucleatum* and *P. fokini* n. sp. (Figure 7). The cells of the *P. fokini* n. sp. strains were neither the biggest nor the smallest among the cells of all 33 analyzed strains. Their CVs had 6–8 collecting canals and 1 pore; similar to in the strains of the group we identified as *P. multimicronucleatum*. The MICs in *P. fokini* n. sp. cells gravitated to the MAC, and this feature was shared by the strains of one of the subgroups of *P. multimicronucleatum*, while the representatives of the two other subgroups had MICs free in the cytoplasm. The oral groove of all strains extended slightly beyond the middle of the cell starting from the cell equator, and the cytoproct of the strains of *P. fokini* n. sp. was shifted to the posterior end of the cell compared with the strains of *P. multimicronucleatum*. Autogamy was not observed in any strain of both groups in daily re-isolated lines. However, in mass cultures of two *P. multimicronucleatum* strains (OmN-1, L72-1), we noted some cells with fragmented MACs. We figured out that moderate selfing (intrastrain conjugation) started in these strains after 10 days of abundant feeding followed by 4 days of starvation. Some exconjugants survived, and in daily re-isolation experiments starting from an exconjugant cell, we found that the next round of selfing could occur after approximately 20 vegetative divisions. Two strains of *P. fokini* n. sp., namely PP2-1 and T42-1, were characterized by very intense, almost total selfing achieved by mild starvation following intense growth. The exconjugants were never viable, not dividing or dying after the first vegetative division. There were four MAC anlagen in the exconjugant cells of both species.

## 4. Discussion

### 4.1. Characteristics of Micronuclei and Paramecium Systematics

The issue of whether “morphology or molecules” should primarily be taken into account within systematics of different groups of protists [43,44], and in particular, in ciliates [45,46,47] has been under debate for the last decade. Currently, molecular data are favored and seem to give more detailed results, although morphological traits are still extremely valuable and provide important complementary data [48,49,50,51]. While general appearance (i.e., cell shape and size) allows for quick and rough species assignment in *Paramecium*, type and number of MICs, along with the morphology of contractile vacuoles, are considered the most important discriminating fine traits of these ciliates [16]. Indeed, for most species of *Paramecium*, the characteristics of MICs and CVs by themselves or in combination are sufficient for identification [16]. However, for example, relationships between *P. woodruffi*, *P. nephridiatum*, and *P. calkinsi* from the Cypriostomum subgenus cannot be faithfully resolved using only these morphological markers [16,17,52]. The same concerns the group of species in the focus of the present paper.

The contractile vacuoles of all species of Paramecium *s. str.* subgenus are almost indistinguishable, having from 5 to 9 canals and normally a single pore, thus leaving the MIC characteristics as the key to identification. It was always believed that MICs are small and multiple in *P. multimicronucleatum* [16]. The cells of the cryptic species “*Eucandidatus* P. brazilianum” had just one or two MICs [11]. In the other recently described new species *P. grohmannae,* the MICs were a bit larger and less numerous (single or, less frequently, two) than in *P. multimicronucleatum* [22]. However, we showed that the number of MICs varies mostly from one to three across *P. multimicronucleatum*, *P. fokini* n. sp., and *P. lynni* n. sp. Moreover, in our selection, one-third of the strains of “classical” *P. multimicronucleatum* had a single MIC, in contradiction to the species name.

The number of MICs appears constant for a given strain, in agreement with the observations of Wichterman [4]. The MIC morphology is variable in *P. multimicronucleatum*-like species, from typical for *P. multimicronucleatum* vesicular MICs and the non-typical vesicular MICs of “*Eucandidatus* P. brazilianum” [11] to previously undocumented for Paramecium *s. str.* endosomal type in *P. grohmannae* [22], and even to somewhat called “small compact MIC” in “*Eucandidatus* P. germanicum” [11]. In our selection of *P. multimicronucleatum* and *P. fokini* n. sp. strains, many variations of MICs were represented. In general, the MICs of all strains were of small size with a dense chromatin body surrounded by a well-visible nuclear envelope. An evident vesicle in the middle of the chromatin body was characteristic for some strains, while in other strains, the MICs looked similar but lacked a vesicle. Many microscopical observations make us think that the vesicle in the MICs is characteristic for *P. multimicronucleatum* and related species but may not always be detected depending on which side the MIC is turned. When the vesicle is not visible or absent, such MICs may be taken as the nuclei of an endosomal type. Few strains also had MICs with stacked chromatin fibers instead of the chromatin body, thus resembling a “chromosomal” type of nuclei. Other morphological characteristics, such as the position of MICs relative to the MAC in a cell or cytoproct location being a bit shifted in *P. fokini* n. sp. strains compared with *P. multimicronucleatum,* are even more difficult to detect. All of these minor differences were not noticed as species-discriminating features before. One not having a sufficient number of strains for comparative observations or just simply lacking visual expertise in *Paramecium* would hardly pay attention to such peculiar morphological deviations. At the same time, just an unusual type and number of MICs might be considered a feature sufficient to designate it as a species, as happened with *P. grohmannae* [22] and “*Eucandidatus* P. brazilianum” [11].

In summary, most of the species belonging to the Paramecium *s. str.* subgenus have MICs that are tiny, being less than 4.5 µm in diameter. “Classical” vesicular MICs characteristic for the *P. aurelia* complex representatives closely resemble smaller vesicular MICs of the *P. multimicronucleatum* strains but do not look the same. Some strains have unpronounced vesicles in their MICs, similar to the nuclei of endosomal type. The strains of *P. lynni* n. sp. had the new “fried egg” type of MIC (see Figure 6), where some chromatin was still present at the periphery of the nucleus between the distinct chromatin body and the nuclear envelope. Such a MIC could also probably be judged as a “small compact” MIC mentioned in the “*Eucandidatus* P. germanicum” description [11], if the envelope was not noticed on live material. Only *P. caudatum* can be easily recognized by its single compact large MIC, and *P. jenningsi* and *P. sonneborni* have two vivid MICs of the “chromosomal” type per cell [16,53,54]. At the same time, based on the recent phylogenomic analysis, the latter two species should be considered members of the *P. aurelia* complex [20]. We also report chromosomal-like MICs in some *P. multimicronucleatum* strains (Figure 6). Therefore, there is no continuous evolutionary row of MIC morphologic variants within the Paramecium *s. str.* subgenus, but instead, in each branch, the generative nucleus may have a different structure, leading to occasional similarities. This indicates that the molecular bases for such morphological characteristics are much more complex than previously expected and could not be correlated with phylogenetic patterns. Variability in the MIC morphology across the studied species prevents us from considering it as a discriminative criterion in species identification, even though it remains one of the key morphological features.

### 4.2. Dubious Paramecium Species

**Figure 8:**
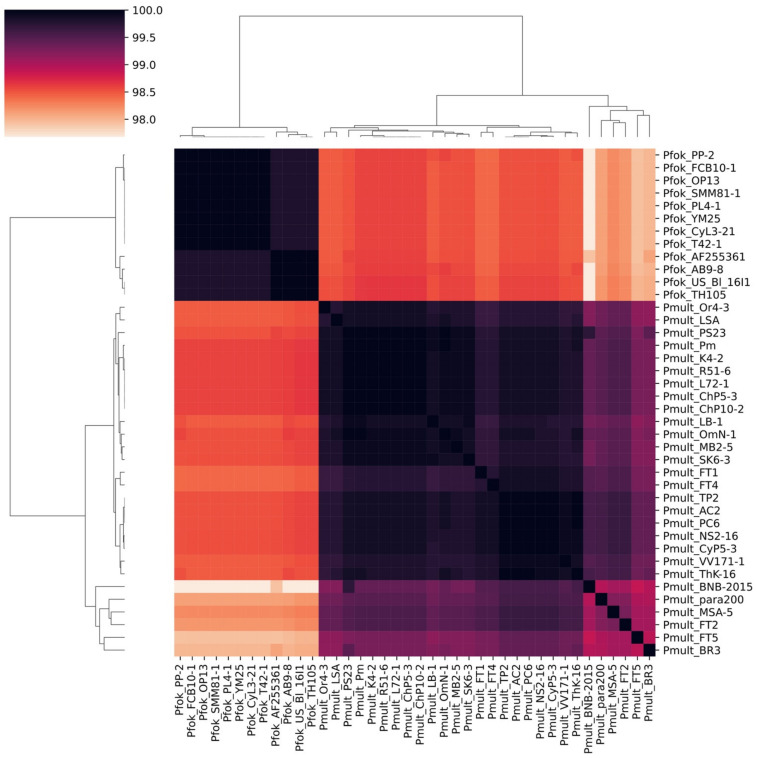
Heatmap based on the matrix populated with the percent identities of pairwise alignments of 18S rRNA gene sequences from *P. multimicronucleatum* and *P. fokini* n. sp. dataset. Pmult stands for *P. multimicronucleatum*, Pfok stands for *P. fokini* n. sp., Pmult_BNB-2105 corresponds to *P. grohmannae*, and Pmult_BR3 corresponds to “*Eucandidatus* P. brazilianum”.

We obtained three molecular phylogenetic trees utilizing the three marker sequences most frequently used in ciliate phylogenetics. We analyzed all available data in the GenBank sequences of the 18S rRNA gene, the ITS region, and the COI gene belonging to the species of the Paramecium *s. str.* subgenus, excluding only identical sequences from the same populations. In general, we obtained almost the same clusters of strains in all three constructed molecular phylogenies, and the COI gene expectedly provided the best resolution, while the 18S rRNA gene and the ITS region were more conserved. The 18S rRNA gene and the ITS region trees had very similar configuration (Figure 2 and Figure 3). All species were monophyletic. Three clusters could be identified within *P. multimicronucleatum*: two clusters within its sister species *P. fokini* n. sp., while no subdivision was observed within *P. lynni* n. sp. In all clusters, the strains originating from remote parts of the world were represented together. Since the morphological criteria in the studied group of species appeared to be obscure, the phylogeny inferred from the 18S rRNA gene came to the forefront to clarify the correct rank of two new taxa described within *P. multimicronucleatum* in the last five years, namely “*Eucandidatus* P. brazilianum” [11] and *P. grohmannae* [22]. The strains representing both new taxa had Brazilian origins. The new species descriptions were based first on the non-canonical vesicular or endosomal types of 1–2 MICs in the strains representing these species (see above), while *P. multimicronucleatum* was known to have multiple vesicular MICs. Our findings of MICs with hardly detectable vesicles in *P. fokini* n. sp. and evidence that some *P. multimicronucleatum* and *P. fokini* n. sp. strains may have a single MIC made it obvious that these features are not so unique within *P. multimicronucleatum*-like strains. Thus, they are not sufficient to nominate *“Eucandidatus* P. brazilianum” and *P. grohmannae* as separate species. The molecular phylogeny inferred by us from the 18S rRNA gene sequences trimmed according to a short available sequence of *P. grohmannae* (1220 bp) confirmed the position of *P. grohmannae* inside of the classical *P. multimicronucleatum* branch, which is visualized in the heatmap on Figure 8. Moreover, *P. grohmannae* falls into one cluster with “*Eucandidatus* P. brazilianum” and several strains from Pakistan and India, forming a small branch within *P. multimicronucleatum* (Figure 8). Unfortunately, the ITS region or the COI gene had not been sequenced either for *P. grohmannae*, “*Eucandidatus* P. brazilianum” or any other strain from this cluster, so it was not possible to determine the position of strains from this subgroup in molecular phylogenies based on other markers. We did not have any strain from this cluster in our collection to check its morphology and to compare it with the descriptions of “*Eucandidatus* P. brazilianum” and *P. grohmannae*. However, a comparison of the 18S rRNA gene sequences confirms that the intraspecific clusters of *P. multimicronucleatum sensu stricto* and, even more so, individual strains from these clusters, even if morphologically deviant, should not be considered as separate species. These may represent, for example, different syngens, which cannot be proved without mating tests.

### 4.3. Paramecium lynni n. sp., the “Stealth Species”

Five strains from the examined selection represented the new morphological species of *Paramecium* phylogenetically separated from all other species. These strains initially were evaluated as “weird *P. multimicronucleatum*” as their cells did not have typical cigar shape but looked more olivary and even plump. Their somatic nuclei also looked roundish. Their MICs were a bit bigger than vesicular MICs of *P. multimicronucleatum*; the vesicle in MIC was not always pronounced; and after Feulgen procedure, the chromatin “halo” was visible between the brightly stained chromatin body and the nuclear envelope. In our opinion, this kind of MIC can be considered a new “fried egg” type. The number of MICs was 2 or 3, and the MICs were not associated with the MAC but scattered in the cytoplasm. All of the above mentioned differences between these strains and *P. multimicronucleatum*, even if discernible, by themselves might not matter, as in *Paramecium* certain variations at morphospecies level are quite common. This was probably the reason why this species has not been described until now, as it seems that it is not rare. In any case, a molecular phylogenetic analysis was crucial to consider this group of strains as a separate species. In all three obtained molecular phylogenetic trees, the five strains studied formed a distinct branch remote from *P. multimicronucleatum*. In the trees inferred from the 18S rRNA gene (Figure 2) and the ITS region (Figure 3), this branch appeared as a sister clade to *P. caudatum*. Interestingly, it also included the strain representing a cryptic species “*Eucandidatus* P. germanicum” [10]. This branch appeared in the same position in the giant COI gene tree, which utilized all available GenBank *Paramecium* COI gene sequences [24], where it formed a sister clade with the sequence of “*Eucandidatus* P. germanicum”. In the COI gene tree obtained by us, the sequence of “*Eucandidatus* P. germanicum” occupies its own branch immediately prior to the divergence of the group of strains in question, which may be due to an unintentional miss of some sequences from the outgroup in our analysis.

Nevertheless, in the COI gene phylogeny, *P. lynni* n. sp. branched basally to all morphological species of Paramecium *s. str.* subgenus before *P. caudatum* divergence from *P. multimicronucleatum* and the *P. aurelia* complex. This clade is also distinguishable in the same basal position in the previously published molecular phylogenies [11,12] and can be traced by the sequences used both in those work and in our study. Unfortunately, in one of those works [12], the published COI gene tree completely lacks *P. caudatum* sequences, so the strains forming this branch were mistakenly assigned to *P. multimicronucleatum*. Later, that mistake might have misled the other group of authors [11], who still assumed that these strains could represent at least a new cryptic or even morphological species, as the genetic distance between them and *P. multimicronucleatum* was too big. All molecular phylogenetic data unequivocally indicate that the strains from this group represent a separate species, which should be ranked as a true morphological species having certain morphological distinctive features, such as the cell shape and specific kind of MIC. Moreover, by molecular phylogenetic data, it groups closer to *P. caudatum* but has no morphological similarity with it. It is, probably, broadly distributed in nature at least in Eurasia, as the strains in our study originated from the environs of Saint Petersburg and from Western Siberia, while in GenBank, we found the sequences definitely belonging to *P. lynni* n. sp. strains from the northwest of Russia, Central Siberia and China. We named this new species *Paramecium lynni* n. sp. in honor and memory of Denis Lynn, the prominent Canadian ciliate biologist and founder of *Paramecium* molecular phylogenetics.

### 4.4. Paramecium fokini n. sp., the Cryptic Species within Paramecium multimicronucleatum Clade

There are two distinct branches in the *P. multimicronucleatum* clade in all existing molecular phylogenies of *Paramecium* utilizing the 18S rRNA gene, the ITS region, and the COI gene sequences [10,11,12,31]. According to the 18S rRNA gene sequence comparison, the identity among the strains from the two groups is not more than 98.6% (see Figure 8), which is much less than the similarity among the sibling species of the *P. aurelia* complex (minimum 99.5%) or between the sister morphospecies *P. aurelia* and *P. jenningsi* (minimum 99.35%). It was supposed earlier that these two branches may represent two cryptic species [11]. However, there were no attempts to compare the morphology and physiology of strains from these branches, so the question remained open. We were the first to perform such a comparison, and we found very slight, if any, morphological differences between the strains of these two groups. A certain variability in MIC appearance was characteristic for the strains of both species, though in general, their MICs should be attributed to the vesicular type. While twelve strains of *P. multimicronucleatum* were characterized by MICs that might be located either in proximity to MAC or freely in cytoplasm, all seven strains of *P. fokini* n. sp. had MICs always positioned close to the MAC. According to the rule coined by W. Foissner, “we classify new species as such only when populations can be separated from their nearest relatives by at least one distinct (nonmorphometric) morphological character” (cited after [55]). This difference would be sufficient to claim that *P. multimicronucleatum* and *P. fokini* n. sp. represent two distinct species. However, as we discussed above, the MIC morphology and other characteristics are widely variative within and among these species and cannot be considered to be used as the all-sufficient species-discriminating characteristic. In general, it would not be possible to distinguish the strains of one species from the strains of the other by means of morphological comparison.

Interestingly, selfing was observed in some strains from both groups. However, this intrastrain mating was always synchronously involving almost all cells of the culture in two strains of *P. fokini* n. sp., while in two strains of *P. multimicronucleatum*, it was much less intense. Exconjugants of *P. fokini* n. sp. strains were never viable, so selfing was a “dead-end” action for such cultures. At the same time, it was possible to obtain clonal cultures from about 50% of exconjugants of *P. multimicronucleatum* strains. The mechanisms underlying these two modes of selfing are probably different. It is important to note that several other strains from both species were tested for the occurrence of the intrastrain conjugation, but no signs of sexual reactivity were noticed. Thus, we suppose that the ability of some *P. multimicronucleatum* and *P. fokini* n. sp. strains to proceed regularly through selfing could be a strain-specific characteristic.

Nevertheless, molecular phylogenetic data witnessed that two groups of *P. multimicronucleatum* strains, in fact, should be treated as two cryptic species. To visualize the difference between representatives of *P. multimicronucleatum* and *P. fokini* n. sp., we applied FISH using specific probes. The 18S rRNA-targeted probes have been successfully applied to identify and count amoebae [56] and flagellates [57,58] in mixed environmental samples. A possibility to design FISH probes specifically recognizing cryptic species in ciliates was approved as an approach to identify three morphologically identical species of *Euplotes* sharing the same environments [59] and two sibling species in *Stylonychia* [60]. We succeeded in designing one FISH probe recognizing only cells of the strains of *P. multimicronucleatum* and giving negative results when applied to cells of *P. fokini* n. sp. strains. All of these data together allow us to suggest that a rank of separate species should be assigned to *P. multimicronucleatum* and *P. fokini* n. sp. The strains of group I satisfy the existing diagnosis of *P. multimicronucleatum*, so this name should be kept for group I. We named the new species represented by the strains of group II *Paramecium fokini* n. sp. in honor of Sergei Fokin, the recognized Russian specialist in *Paramecium* biology.

### 4.5. New Insights into Biogeography of Paramecium

*Paramecium multimicronucleatum* is one of the most common *Paramecium* morphospecies distributed worldwide [16]. Since representative collections of *P. multimicronucleatum* never existed, up until now there were no data showing geographic patterns of the occurrence of five syngens reported for this species [4,61] as well as their phylogenetic interrelations. Now, with the subdivision of *P. multimicronucleatum* into two separate cryptic species, it becomes even more questionable how many syngens exist within each of them. Syngens distribution has been assessed earlier for two morphological species of *Paramecium*. In *P. bursaria,* there are five known syngens [14,62]; two of those are met exclusively across Europe and up to Eastern Siberia, while the third is common for the Russian Far East, China, Japan, North America, and South America but was rarely registered in Europe [14]. For the remaining two syngens there are just a few strains known, so it is not possible to make any firm conclusions. Instead, all sibling species of the *P. aurelia* complex are either very rare or, if widespread, are represented all over the world, though with certain climatic preferences. For example, *P. biaurelia* occurs frequently only in moderate climate zones [63,64], while *P. sexaurelia* tends to occur in low latitudes [65]. Thus, the known principle “everything is everywhere” cannot be directly applied to *Paramecium*, though the geographic zones inhabited by many *Paramecium* species continue to expand with extensive sampling in previously unexplored regions [19,54,66]. Twenty strains of *P. multimicronucleatum s. str.* from all over the world were analyzed in our study. In addition, there are about 50 more sequences in GenBank, making a sufficient selection to address the biogeography of this species.

The majority of the phylogenetic clusters detected by all three utilized markers, with a few exceptions, included strains from very geographically remote populations, in accordance with the data from other authors [12,67,68]. At the same time, the sequences from strains sharing an origin could belong to different clusters, for example, strains from Mexico in our work or strains collected in China [68]. Thus, the level of polymorphism in local populations in some cases may be comparable with the worldwide diversity level, and at the same time, the strains from very remote locations may belong to the same haplotype. It is difficult to imagine that populations from different continents might have the same founders. Since *Paramecium* cannot form cysts, the most plausible explanations could be extensive migration of paramecia through the systems of waterbodies during seasonal floods or hijacking waterfowl or aquatic insects, though both hypotheses do not provide solutions at least for transoceanic spread.

As it is quite plausible that the intraspecific groups revealed by the molecular phylogenetic approach correspond to *P. multimicronucleatum* syngens, it was tempting to determine if the strains from the same group share geographic origins or were at least collected on the same continent or geographic zone. The COI gene has proven to be the best marker for dissecting intraspecific polymorphism in *Paramecium* [42,67,69]. According to the COI phylogeny, we detected at least three subgroups within *P. multimicronucleatum s. str.* (Figure 4), and there should be more, since strains from the 18S rRNA gene tree clade including *P. grohmannae* and “*Eucandidatus* P. brazilianum” were not represented in the COI gene dendrogram. Two of the three identified groups can be further subdivided into smaller clusters (Figure 4), showing that divergence is ongoing. The strains from all continents appeared to be present in two subgroups: subgroup 1 included strains from China, Thailand, India, Europe, Brazil, and Hawaii; subgroup 2 consisted of the strains originating from Mexico, Italy, Moldova, China, and Japan. The most geographically consistent was subgroup 3, which showed at least three further diverging lineages, and all of them included only strains from Mexico and the USA, with a single exception: one strain from Portugal. We had on hand several strains from this branch, which were characterized with occasional intrastrain conjugation (selfing). Such sexual behavior is not common in *Paramecium*, and selfing is mostly considered a rare event when some cells within strain randomly change their mating type and become able to mate with other cells from the same strain. The control of mating types in *P. multimicronucleatum* is poorly understood. It is considered that the mating types are stochastically determined in karyonides after nuclear reorganization following sexual process [4,70]. In some *P. multimicronucleatum* syngen 2 strains, the mating types switched daily according to circadian rhythms, leading to selfing in the transition phase [71]. It might be that subgroup 3 in the COI gene phylogeny corresponds to syngen 2 then. It has been shown also [71] that the sexual immaturity period in *P. multimicronucleatum* lasts for 60–90 vegetative cell fissions, and early maturity, within 20 fissions of conjugation, is a result of MAC regeneration or aberrant nuclear reorganization. Exconjugant clones in our study were becoming sexually reactive again after 20 cell divisions. Thus, the inability of normal nuclear rearrangements phenotypically can be detected as frequent selfing and, possibly, is also a genetic feature of certain *P. multimicronucleatum* strains.

Apparently, there are also two groups within *P. fokini* n. sp., and while one of them is clearly cosmopolitan, the other includes only strains with a known or supposed origin from the USA. Thus, it cannot be excluded that some such intraspecific groups, likely syngens, are limited to North and Central America, but this hypothesis should be treated with caution, requiring more confirmations with a lot of new findings. However, the data obtained by us are enough to suppose that the geographic factor may contribute to initial speciation in *P. multimicronucleatum* and *P. fokini* n. sp.

### 4.6. The New Paramecium Species Formal Descriptions

Paramecium lynni Melekhin, Lebedeva, and Potekhin, 2022 n. sp. taxonomic summary.

Differential diagnosis. Body size ranged 133.7 ± 13.1 × 31.9 ± 5.1 µm (fixed by Bouin solution); rather wide, dorso-ventrally flattened cells, both ends of the cell seem blunt; the number of cilia rows is 64 ± 8; the cytoproct localized midway between the buccal overture and the posterior end of the cell; two contractile vacuoles with one pore each and 6–8 collecting canals; 1–3 fried egg-like micronuclei 3.55 ± 0.46 µm in diameter localized freely in the cytoplasm; autogamy was never observed; freshwater, bacterivorous.

Type locality. The pond in Sestroretsk, Saint Petersburg, Russia; 60°05′49″N, 29°57′36″E.

Type slides. Several holotype and paratype slides have been deposited in the collection of microscopical slides of the Department of Invertebrate Zoology, St. Petersburg State University, Russia.

Type culture. The type strain SD11-9 and other strains of the species are maintained in the RC CCM culture collection (World Data Centre for Microorganisms, RN 1171) of Saint Petersburg State University, Saint Petersburg, Russia.

Sequence availability. The 18S and ITS rRNA gene nucleotide sequences and the COI gene of the type strain were deposited in the NCBI GenBank database under accession numbers OM200751 and OM401926.

Zoobank Registration LSID: 11084765-E304-48F2-AA8E-84ABAF265F94.

Further remarks. Related species: *Eucandidatus* Paramecium germanicum. Endosymbionts not described so far.

*Paramecium multimicronucleatum* Powers and Mitchell, 1910 amended taxonomic summary.

Differential diagnosis. Body size ranged 153.4 ± 11.9 × 31.6 ± 4.1 µm (fixed by Bouin solution); dorso-ventrally flattened cells with narrowed posterior end; the number of cilia rows vary 73 ± 8; the cytoproct extended slightly between the cytostome and the posterior end of the cell; two contractile vacuoles with one pore each and 5–8 collecting canals; 1–3 vesicular MICs (sometimes vesicle is hardly visible) localized freely in cytoplasm or gravitated to MAC; autogamy was never observed; some strains are characterized by intrastrain conjugation after mild starvation; the progeny is poorly viable; four MAC anlagen formed after sexual process; freshwater, bacterivorous.

Type culture. Since the type strain described by Powers and Mitchell has been lost, we suggest considering strain CyP5-3 as a typical representative of the species. Strain CyP5-3 and other strains of the species are maintained in the RC CCM culture collection (World Data Centre for Microorganisms, RN 1171) of Saint Petersburg State University, Saint Petersburg, Russia.

Sequence availability. The 18S and ITS rRNA gene nucleotide sequences and the COI gene of CyP5-3 strain were deposited in the NCBI GenBank database under accession numbers OM200731 and OM401905.

Reported endosymbionts: “*Candidatus* Trichorickettsia mobilis” [72,73], “*Candidatus* Gortzia shahrazadis” [74].

*Paramecium fokini* Melekhin, Nekrasova, Petroni, and Potekhin, 2021 n. sp. taxonomic summary.

Differential diagnosis. Body size ranged 155.6 ± 13.9 × 27.6 ± 4.8 µm (fixed by Bouin solution); dorso-ventrally flattened cells reminiscent of classical *P. multimicronucleatum* form with narrowed posterior end; the number of cilia rows is 67 ± 8; the cytoproct is shifted to the posterior end of the cell; two contractile vacuoles with one pore each and 6–8 collecting canals; 1–3 vesicular (sometimes vesicle is not pronounced) MICs gravitate to MAC; autogamy was never observed; some strains are characterized by intrastrain conjugation after mild starvation; the progeny is not viable; four MAC anlagen formed after sexual process; freshwater, bacterivorous.

Type locality. The ditch in Pochap village, Pskov region, Russia; 58°37′01″N 29°00′08″E.

Type slides. Several holotype and paratype slides have been deposited in the collection of microscopical slides of the Department of Invertebrate Zoology, Saint Petersburg State University, Russia.

Type culture. The type strain PP-2 and other strains of the species are maintained in the RC CCM culture collection (World Data Centre for Microorganisms, RN 1171) of St Petersburg State University, Saint Petersburg, Russia.

Sequence availability. The 18S and ITS rRNA gene nucleotide sequences and the COI gene of the type strain were deposited in the NCBI GenBank database under accession numbers OM200746 and OM401921.

Zoobank Registration LSID: 3509110D-286C-49FF-A46C-6B5537C4A40A.

Further remarks. Cryptic species; the closest related species is *Paramecium multimicronucleatum*; may be discriminated from the latter by FISH; reported endosymbionts: “*Candidatus* Trichorickettsia mobilis” [75].

## 5. Conclusions

In this study, we showed that a thorough comparative morphological analysis enlightened by molecular phylogenetic data allows for finding new morphological species even within a fairly well-known subgenus of *Paramecium*. We described a novel morphological species *P. lynni* n. sp., and we split the *P. multimicronucleatum* morphospecies into two cryptic species, *P. multimicronucleatum s. str.* and *P. fokini* n. sp. Accordingly, when the molecular data contradicted the morphological observations, an analysis of more strains from the same phylogenetic group showed that morphological peculiarities thought to be species-specific in fact were not, thus leading to a rejection of two recently announced species, namely *P. grohmannae* and *Eucandidatus* P. brazilianum. Molecular phylogenetics has good predictive power, as both newly described species were detectable in all previously published trees inferred from sufficient selections of strains. We can expect further descriptions of the new species in *Paramecium*, as at least two more such “phylogenetic groups” were recently reported in its Paramecium *s. str.* [23,76] and Helianter [24] subgenera.

## Data Availability

Not applicable.

## Supplementary Materials

The following supporting information can be downloaded at https://www.mdpi.com/article/10.3390/microorganisms10050974/s1, Figure S1: Discrimination of representatives of *P. multimicronucleatum* and *P. fokini* n. sp. by FISH, Figure S2: Variability of MIC appearance in the cells of *P. multimicronucleatum* strain L72-1, Table S1: Oligonucleotides used in the study, Table S2: Specificity of the FISH probes applied, Table S3: List of the *Paramecium* strains misidentified in GenBank. References [77,78,79] are cited in the Supplementary Materials.
