# Supplementary material for: Cryptic Diversity in Paramecium multimicronucleatum Revealed with a Polyphasic Approach
Source: Microorganisms. 2022 May 5;10(5):974. doi: 10.3390/microorganisms10050974 (PMC9143557; doi:10.3390/microorganisms10050974)
Supplement: Supplementary file 1 [file microorganisms-10-00974-s001.zip › Suppl Table 1.pdf]

Table S1. Oligonucleotides used in the study.

Marker gene	Used for	Name of the oligonucleotide	Sequence (5′-3′)	Reference
18S rRNA	PCR	18SR1513Hypo	TGATCCTTCYGCAGGTTC	[77]
		18SF9	CTGGTTGATCCTGCCAG	[78]
	Sequencing	18SR1052	AACTAAGAACGGCCATGCA	[79]
		18SR536	CTGGAATTACCGCGGCTG	[79]
		18SF783	GACGATCAGATACCGTC	[79]
	FISH	Paramulti	AGAACCAACTGCTAGATAGC	This study
		Parafok	GAACCAGCTAAATAAGTGCC	This study
ITS1-5.8S-ITS2	PCR	18SF919	ATTGACGGAAGGGCACCA	[79]
		28SR671	TAGTTCACCATCTTTCGGG	Unpublished
	Sequencing	FG1400	TTGYACACACCGCCCGTC	[31]
		RGD2	GGTCCGTGTTTCAAGACGGG	[31]
COI	PCR and sequencing	F388dt	TGTAAAACGACGGCCAGTGGWKCBAAAGATGTWGC	[32]
		R1184dt	CAGGAAACAGCTATGACTADACYTCAGGGTGACCRAAAAATCA	[32]
