# Supplementary material for: Cryptic Diversity in Paramecium multimicronucleatum Revealed with a Polyphasic Approach
Source: Microorganisms. 2022 May 5;10(5):974. doi: 10.3390/microorganisms10050974 (PMC9143557; doi:10.3390/microorganisms10050974)
Supplement: Supplementary file 1 [file microorganisms-10-00974-s001.zip › Suppl Table 2.pdf]

Table S2. Specificity of the FISH probes applied.

	Probes	
Paramulti	GCTATCTAGCAGTTGGTTCT	
Parafok		GGCACTTATTTAGCTGGTTC
Species	Target sequence	Target sequence
P. multimicronucleatum	GCTATCTAGCAGTTGGTTCT	GGCTATCTAGCA GTTGGTTC
P. fokini n.sp.	GCACTTATTTAGC TGGTTCT	GGCACTTATTTA GCT GGTTC
P. caudatum	TCAGGCTGCTAATAGGCTCT	GTCAGGCTGCTAATAGGCTC
P. lynni n.sp.	TCAGGCTGTCAATAGGCTCT	GTCAGGCTGTCAATAGGCTC
P. aurelia complex	TCAGTTAGTTAAT TGATTCT	GTCAGTTAGTT AATT GATTC

The matching nucleotides are labeled with colors with respect to the probe.
