# Supplementary material for: Cryptic Diversity in Paramecium multimicronucleatum Revealed with a Polyphasic Approach
Source: Microorganisms. 2022 May 5;10(5):974. doi: 10.3390/microorganisms10050974 (PMC9143557; doi:10.3390/microorganisms10050974)
Supplement: Supplementary file 1 [file microorganisms-10-00974-s001.zip › Suppl Table 3.pdf]

#	ID	Strain name	Given name	Suggested name	Doi of the article / the source	Comments
1	MN264582	G1-2	*Paramecium* sp.	*Paramecium multimicronucleatum*	Unpublished	
2	MN264576	GB4-26	*Paramecium multimicronucleatum*	*Paramecium caudatum*	Unpublished	
3	JF741258	Kr113-3	*Paramecium multimicronucleatum*	*Paramecium caudatum*	https://doi.org/10.1016/j.ympev.2012.01.024	
4	JF741263	TRB101-4	*Paramecium multimicronucleatum*	*Paramecium lynni*	https://doi.org/10.1016/j.ympev.2012.01.024	
5	JF741262	TRB101-1	*Paramecium multimicronucleatum*	*Paramecium lynni*	https://doi.org/10.1016/j.ympev.2012.01.024	
6	MN264581	CB8-2	*Paramecium* sp.	*Paramecium lynni*	Unpublished	
7	MN264579	V105-1	*Paramecium* sp.	*Paramecium lynni*	Unpublished	
8	MT078149	SMM81-1	*Paramecium multimicronucleatum*	*Paramecium fokini*	http://dx.doi.org/10.3390/d12050197	
9	MT078148	T42-1	*Paramecium multimicronucleatum*	*Paramecium fokini*	http://dx.doi.org/10.3390/d12050197	
10	MN264577	AM	*Paramecium multimicronucleatum*	*Paramecium fokini*	Unpublished	
11	JF304189	BR	*Paramecium multimicronucleatum*	*Paramecium fokini*	https://doi.org/10.1016/j.protis.2011.06.006	
12	FJ905144	OLI-Parame-mult-02	*Paramecium multimicronucleatum*	*Paramecium fokini*	https://doi.org/10.1080/14772000903507744	
13	FJ905143	OLI-Parame-mult-01	*Paramecium multimicronucleatum*	*Paramecium fokini*	https://doi.org/10.1080/14772000903507744	
14	MK806287	US_Bl 16I1	*Paramecium multimicronucleatum*	*Paramecium fokini*	https://doi.org/10.1101/688770	
15	mito_mult_M04	M03c4	*Paramecium multimicronucleatum*	*Paramecium fokini*	https://paramecium.i2bc.paris-saclay.fr	
16	KJ755359	BNB-2015	*Paramecium grohmannae*	*Paramecium multimicronucleatum*	No doi	"Description and molecular phylogeny of *Paramecium grohmannae* sp. nov. (Ciliophora, Peniculida) from a wastewater treatment plant in Brazil"
17	AJ548822	BR3	*Paramecium* sp.	*Paramecium multimicronucleatum*	https://doi.org/10.1111/j.1365-294x.2005.02597.x	
18	FR869987	para200	*Paramecium* sp.	*Paramecium multimicronucleatum*	Unpublished	
19	AB252007	YM25	*Paramecium multimicronucleatum*	*Paramecium fokini*	No doi	"Intraspecific genetic divergence of *Paramecium bursaria* and reconstruction of the paramecian phylogenetic tree"
21	AF255361	-	*Paramecium multimicronucleatum*	*Paramecium fokini*	https://doi.org/10.1111/j.1550-7408.2000.tb00069.x	
22	MK595741	US_Bl 16I1	*Paramecium multimicronucleatum*	*Paramecium fokini*	https://doi.org/10.1101/688770	
23	AB252006	TH105	*Paramecium multimicronucleatum*	*Paramecium fokini*	No doi	"Intraspecific genetic divergence of *Paramecium bursaria* and reconstruction of the paramecian phylogenetic tree"
24	HE662765	FT8	*Paramecium caudatum*	*Paramecium* sp.	Unpublished	
25	HE662763	FT6	*Paramecium caudatum*	*Paramecium* sp.	Unpublished	
26	HE662764	FT7	*Paramecium caudatum*	*Paramecium* sp.	Unpublished	
27	KM091236	GO1	*Paramecium* sp.	*Eucandidatus* Paramecium germanicu	https://doi.org/10.1007/s13127-015-0207-9	
28	HM140398	KR-09052101	*Paramecium duboscqui*	*Paramecium* sp.	Unpublished	
29	LN869940	aLdG3	*Paramecium* sp.	*Paramecium bursaria*	https://doi.org/10.1016/j.ejop.2015.12.005	
30	MN264575	G1-2	*Paramecium* sp.	*Paramecium multimicronucleatum*	Unpublished	
31	MN264570	AM	*Paramecium multimicronucleatum*	*Paramecium fokini*	Unpublished	
32	JF741217	OP13	*Paramecium multimicronucleatum*	*Paramecium fokini*	https://doi.org/10.1016/j.ympev.2012.01.024	
33	JF741240	AB9-8	*Paramecium multimicronucleatum*	*Paramecium fokini*	https://doi.org/10.1016/j.ympev.2012.01.024	
34	JF741231	TRB101-4	*Paramecium multimicronucleatum*	*Paramecium lynni*	https://doi.org/10.1016/j.ympev.2012.01.024	
35	JF741230	TRB101-1	*Paramecium multimicronucleatum*	*Paramecium lynni*	https://doi.org/10.1016/j.ympev.2012.01.024	
36	MN264574	CB8-2	*Paramecium* sp.	*Paramecium lynni*	Unpublished	
37	MN264572	V105-1	*Paramecium* sp.	*Paramecium lynni*	Unpublished	
38	JF741226	Kr113-3	*Paramecium multimicronucleatum*	*Paramecium caudatum*	https://doi.org/10.1016/j.ympev.2012.01.024	
39	MN264569	GB4-26	*Paramecium multimicronucleatum*	*Paramecium caudatum*	Unpublished	
