# Supplementary material for: Cryptic Diversity in Paramecium multimicronucleatum Revealed with a Polyphasic Approach
Source: Microorganisms. 2022 May 5;10(5):974. doi: 10.3390/microorganisms10050974 (PMC9143557; doi:10.3390/microorganisms10050974)
Supplement: Supplementary file 1 [file microorganisms-10-00974-s001.zip › Supplementary Figures.pdf]

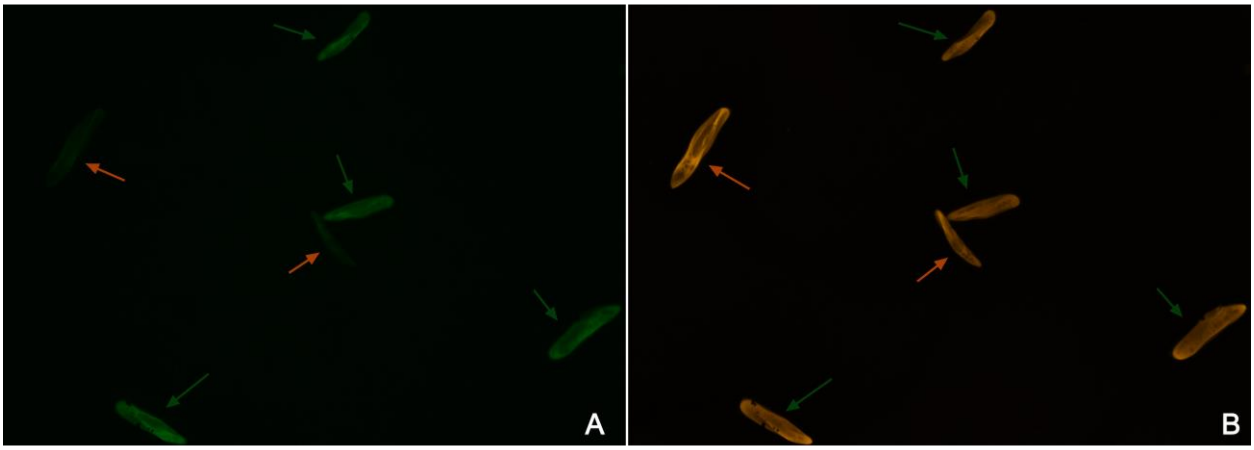

Figure S1. Discrimination of representatives of *P. multimicronucleatum* and *P. fokini* n. sp. by FISH: (A) mixed cells of both species hybridized with Paramulti probe (green signal) specifically designed for group I (*P. multimicronucleatum*); (B) mixed cells of both species hybridized with Parafok probe (orange signal) specifically designed for group II (*P. fokini* n. sp.). Cells of *P. multimicronucleatum* are marked with green arrows, and those of *P. fokini* n. sp. are marked with orange arrows. Paramulti probe appeared to be efficient in exclusively detecting representatives of strains belonging to *P. multimicronucleatum* (cells marked with orange arrows on (A) remain almost invisible), while Parafok bound to cells of both species.

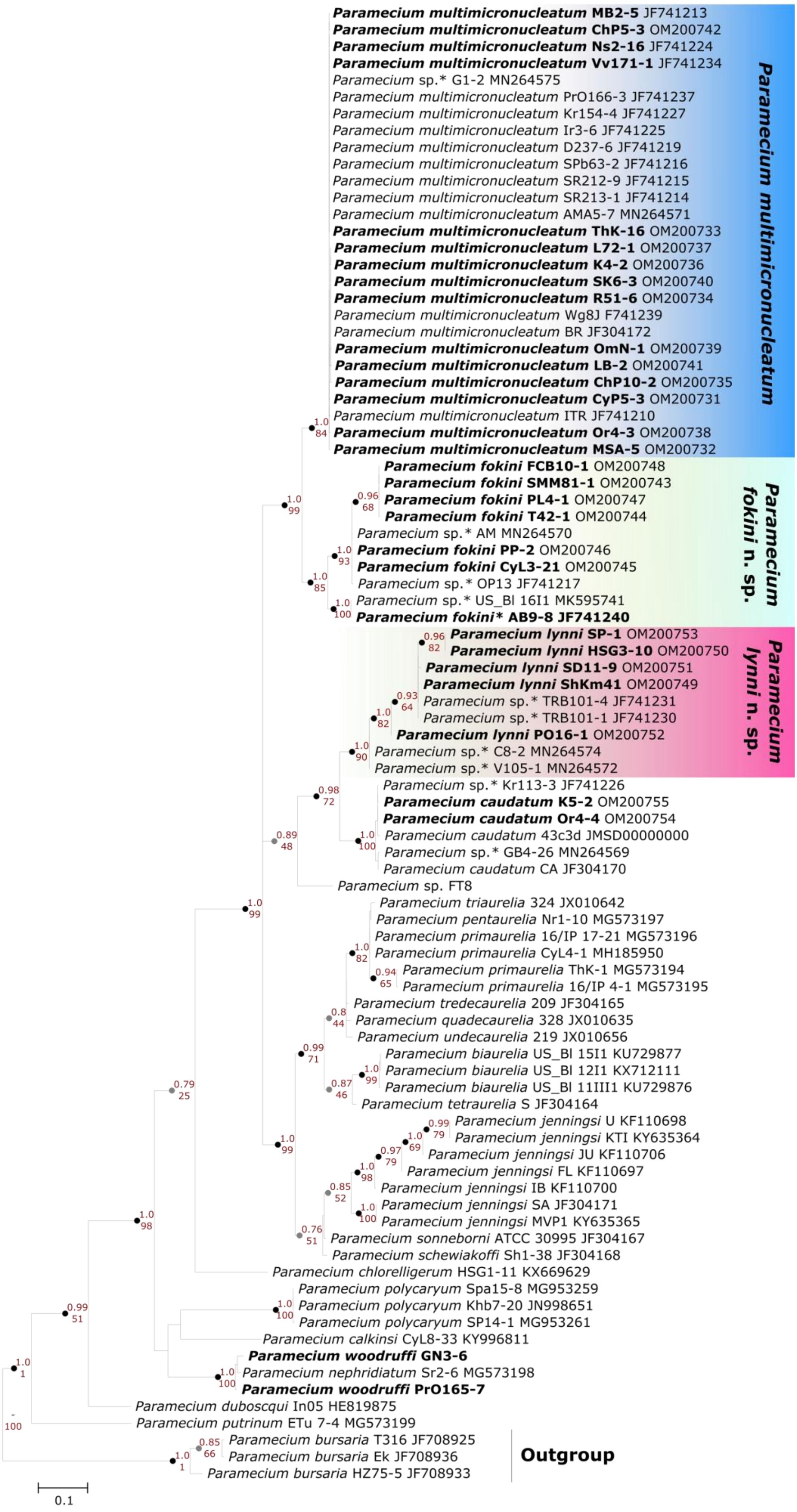

Paramecium multimicronucleatum MB2-5 JF741213
Paramecium multimicronucleatum ChP5-3 OM200742
Paramecium multimicronucleatum Ns2-16 JF741224
Paramecium multimicronucleatum Vv171-1 JF741234
Paramecium sp.* G1-2 MN264575
Paramecium multimicronucleatum PrO166-3 JF741237
Paramecium multimicronucleatum Kr154-4 JF741227
Paramecium multimicronucleatum Ir3-6 JF741225
Paramecium multimicronucleatum D237-6 JF741219
Paramecium multimicronucleatum SPb63-2 JF741216
Paramecium multimicronucleatum SR212-9 JF741215
Paramecium multimicronucleatum SR213-1 JF741214
Paramecium multimicronucleatum AMA5-7 MN264571
Paramecium multimicronucleatum ThK-16 OM200733
Paramecium multimicronucleatum L72-1 OM200737
Paramecium multimicronucleatum K4-2 OM200736
Paramecium multimicronucleatum SK6-3 OM200740
Paramecium multimicronucleatum R51-6 OM200734
Paramecium multimicronucleatum Wg8J F741239
Paramecium multimicronucleatum BR JF304172
Paramecium multimicronucleatum OmN-1 OM200739
Paramecium multimicronucleatum LB-2 OM200741
Paramecium multimicronucleatum ChP10-2 OM200735
Paramecium multimicronucleatum CyP5-3 OM200731
Paramecium multimicronucleatum ITR JF741210
Paramecium multimicronucleatum Or4-3 OM200738
Paramecium multimicronucleatum MSA-5 OM200732
Paramecium multimicronucleatum
Paramecium fokini FCB10-1 OM200748
Paramecium fokini SMM81-1 OM200743
Paramecium fokini PL4-1 OM200747
Paramecium fokini T42-1 OM200744
Paramecium sp.* AM MN264570
Paramecium fokini PP-2 OM200746
Paramecium fokini CyL3-21 OM200745
Paramecium sp.* OP13 JF741217
Paramecium sp.* US_Bl 16I1 MK595741
Paramecium fokini* AB9-8 JF741240
Paramecium fokini n. sp.
Paramecium lynni SP-1 OM200753
Paramecium lynni HSG3-10 OM200750
Paramecium lynni SD11-9 OM200751
Paramecium lynni ShKm41 OM200749
Paramecium sp.* TRB101-4 JF741231
Paramecium sp.* TRB101-1 JF741230
Paramecium lynni PO16-1 OM200752
Paramecium sp.* C8-2 MN264574
Paramecium sp.* V105-1 MN264572
Paramecium lynni n. sp.
Paramecium sp.* Kr113-3 JF741226
Paramecium caudatum K5-2 OM200755
Paramecium caudatum Or4-4 OM200754
Paramecium caudatum 43c3d JMSD00000000
Paramecium sp.* GB4-26 MN264569
Paramecium caudatum CA JF304170
Paramecium sp. FT8
Paramecium triaurelia 324 JX010642
Paramecium pentaurelia Nr1-10 MG573197
Paramecium primaurelia 16/IP 17-21 MG573196
Paramecium primaurelia CyL4-1 MH185950
Paramecium primaurelia ThK-1 MG573194
Paramecium primaurelia 16/IP 4-1 MG573195
Paramecium tredecaurelia 209 JF304165
Paramecium quadecaurelia 328 JX010635
Paramecium undecaurelia 219 JX010656
Paramecium biaurelia US_Bl 15I1 KU729877
Paramecium biaurelia US_Bl 12I1 KX712111
Paramecium biaurelia US_Bl 11III1 KU729876
Paramecium tetraurelia S JF304164
Paramecium jenningsi U KF110698
Paramecium jenningsi KTI KY635364
Paramecium jenningsi JU KF110706
Paramecium jenningsi FL KF110697
Paramecium jenningsi IB KF110700
Paramecium jenningsi SA JF304171
Paramecium jenningsi MVP1 KY635365
Paramecium sonneborni ATCC 30995 JF304167
Paramecium schewiakoffi Sh1-38 JF304168
Paramecium chlorelligerum HSG1-11 KX669629
Paramecium polycaryum Spa15-8 MG953259
Paramecium polycaryum Khb7-20 JN998651
Paramecium polycaryum SP14-1 MG953261
Paramecium calkinsi CyL8-33 KY996811
Paramecium woodruffi GN3-6
Paramecium nephridiatum Sr2-6 MG573198
Paramecium woodruffi PrO165-7
Paramecium duboscqui In05 HE819875
Paramecium putrinum ETu 7-4 MG573199
Paramecium bursaria T316 JF708925
Paramecium bursaria Ek JF708936
Paramecium bursaria HZ75-5 JF708933
Outgroup
0.1

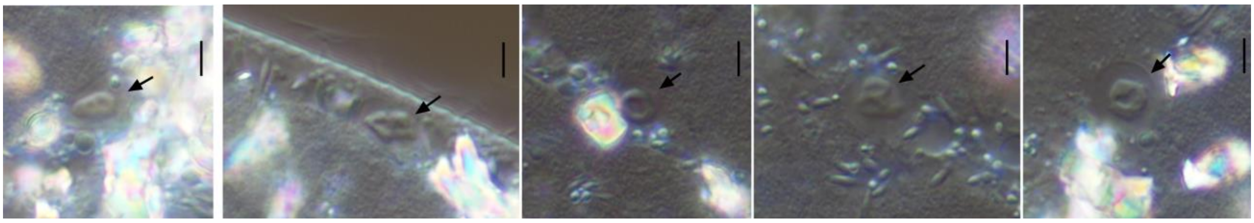

Figure S2. Variability of MIC appearance in the cells of *P. multimicronucleatum* strain L72-1. DIC microscopy. Micronuclei are marked with the arrows. Scale bar is 2,5 mkm.
